# Integrative Analyses of Transcriptomes and Metabolomes Reveal Associated Genes and Metabolites with Flowering Regulation in Common Vetch (*Vicia sativa* L.)

**DOI:** 10.3390/ijms23126818

**Published:** 2022-06-19

**Authors:** Qiang Zhou, Yue Cui, Rui Dong, Dong Luo, Longfa Fang, Zhibiao Nan, Zhipeng Liu

**Affiliations:** 1 State Key Laboratory of Grassland Agro-Ecosystems, Key Laboratory of Grassland Livestock Industry Innovation, Ministry of Agriculture and Rural Affairs, Engineering Research Center of Grassland Industry, Ministry of Education, College of Pastoral Agriculture Science and Technology, Lanzhou University, Lanzhou 730020, China; zhouq2013@lzu.edu.cn (Q.Z.); cuiyue0719@163.com (Y.C.); luod@lzu.edu.cn (D.L.); fanglf@lzu.edu.cn (L.F.); zhibiao@lzu.edu.cn (Z.N.); 2 College of Ecology, Lanzhou University, Lanzhou 730000, China; 3 Department of Grassland Science, College of Animal Science, Guizhou University, Guiyang 550025, China; rdong@gzu.edu.cn

**Keywords:** *Vicia sativa* L., transcriptome, metabolome, flowering, salicylic acid

## Abstract

As an important source of protein for livestock and human consumption, *Vicia sativa* is cultivated worldwide, but its seed production is hampered at high altitudes because of the short frost-free period. Flowering represents the transition from a vegetative to a reproductive period, and early flowering benefits plant seed production at high altitudes. However, the molecular mechanisms of flowering regulation in *V. sativa* remain elusive. In the present study, two *V. sativa* accessions with different flowering characteristics were used: Lan3 (early-flowering) was cultivated by our laboratory, and 503 (late-flowering) was selected from 222 *V. sativa* accessions after three years of field experiments. The shoot samples (shoot tip length = 10 cm) of these two accessions were collected 63, 70, and 77 days after sowing, and the molecular regulatory mechanism of the flowering process was identified by integrative analyses of the transcriptomes and metabolomes. Kyoto Encyclopedia of Genes and Genomes enrichment showed that the synthesis and signal transduction of plant hormone pathways were the most enriched pathways in 4274 differentially expressed genes (DEGs) and in 259 differential metabolites between Lan3 and 503. Moreover, the contents of three metabolites related to salicylic acid biosynthesis and the transcription levels of two DEGs related to salicylic acid signal transduction in Lan3 were higher than those in 503. Further verification in various accessions indicated that salicylic acid metabolism may be involved in the flowering regulation process of *V. sativa*. These findings provide valuable information for understanding the flowering mechanism and for promoting breeding research in *V. sativa*.

## 1. Introduction

Flowering is a central event in the plant life cycle and represents the transition from a vegetative to a reproductive period [1,2], and flowering time is an important determinant of harvesting time. Different from agricultural production, the above-ground part of the plant is the harvest target in forage production. Early flowering contributes to plant seed production and offspring reproduction, but it affects the above-ground biomass of forages. Interestingly, early flowering can also improve the year-round production of some perennial forages by increasing the clipping frequency [1], such as in the case of alfalfa and sorghum–sudangrass hybrids. Therefore, understanding the molecular mechanism behind flowering plays an important role in forage molecular design breeding.

As an important source of protein for livestock and humans, common vetch (*Vicia sativa* L.) is cultivated worldwide and is commonly used in methane biofuel production and in health-promoting foods [3]. *V. sativa* is an annual self-pollinating plant, and it is a semi-erect climbing plant (at the end of each compound leaf is a tendril) that uses adjacent vegetation for support. It is noteworthy that inconspicuous pea-shaped flowers of *V. sativa* grow in the area between the stems and leaf petioles (leaf axils), and they occur in pairs and on flower stalks (peduncles) that are 2–6 mm in length [4]. *V. sativa* can be planted all year round, and the specific time depends on the planting location. In Serbia (Novi Sad) and the Qinghai-Tibet plateau, *V. sativa* are sown at the end of March and April, respectively [5,6]; in northwest Syria, Mexico (Zacatecas), and the Mediterranean coast of Turkey, they are usually sown in November or December [7,8,9]; in central Turkey, they can be sown either in spring or in autumn [10]. In addition, the flowering period of *V. sativa* varies according to climatic circumstances and its own characteristics. For example, a total of 45 *V. sativa* accessions were grown over four cropping seasons in a Mediterranean environment in northwest Syria, and these accessions varied (*p* < 0.05) in days to flowering (78–96 days) [8]; on the Mediterranean coast of Turkey, the days to flowering of another 150 *V. sativa* accessions ranged from 113 to 162 days [7].

*V. sativa* has been successfully domesticated and extensively cultivated in the Qinghai-Tibet subalpine/alpine as the most important sources of crude protein for livestock [11]. However, at high altitudes, frost caused by a shorter frost-free period may damage the flower buds or blooming flowers of plants at the period of bloom, reducing flower bud survival and limiting seed production [12,13,14]. A previous study reported that the vegetative organs tolerated on an average 2–5 K lower freezing temperatures than the reproductive structures in six common European high alpine plant species, and stigma, style, and the flower stalk/peduncle were the most frost-susceptible reproductive structures [15], and similar results were reported in blueberry and apricot [16,17]. It is important to note that the frost resistance of reproductive tissues during different stages of development varies according to species. For example, frost resistance of the flower stalks/peduncles increased significantly from b2 (the corolla is still covered by the sepals) to the f1 (early fruit development) stage in *Ranunculus glacialis* and *Saxifraga bryoides*, and it unchanged or tended to decrease in *Cerastium uniflorum*, *S. caesia*, *S. moschata*, and *Silene acaulis*; frost resistance of the stigma and style tended to increase from b2 to a (corolla open) stage in all species except for *S. acaulis* [15]. Reducing the probability of exposure to frost has clear benefits for the survival of flower buds and flowers [12]; thus, identifying the molecular mechanisms of flowering time control will help cultivate *V. sativa* that is suitable for growth and reproduction in high-altitude areas. The molecular mechanisms that are responsible for flowering in *V. sativa* remain elusive, but our lab has explored the floral transcriptome to examine a genome-scale genetic model of the zygomorphic flower of *V. sativa* [18]. Our comparative analysis of vetch and *Arabidopsis* showed that the vetch flowers conform to a strict ABC model. We analyzed the evolution and expression of the TCP gene family in vetch at a whole-genome level, and several unigenes specific to three different vetch petals, which might offer some clues toward elucidating the molecular mechanisms underlying floral zygomorphy.

Recent studies have shown that flowering is controlled by environmental and endogenous signals [19,20]. As one of the most important environmental factors, the effect of the photoperiod on flowering has been well characterized in the model plant *Arabidopsis thaliana*. Three feedback suppression loops are involved in the *Arabidopsis* core oscillator: central, morning, and evening [21]. The central loop contains *LATE ELONGATED HYPOCOTYL* (*LHY*), *CIRCADIAN CLOCK ASSOCIATED 1* (*CCA1*), and *TIMING OF CAB EXPRESSION 1* (*TOC1*)/*PSEUDO-RESPONSE REGULATOR 1* (*PRR1*); the morning loop consists of *LHY*, *CCA1*, *PRR9*, and *PRR7*; and the evening loop is mainly composed of *TOC1* and *GIGANTEA* (*GI*). Apart from *Arabidopsis*, research into photoperiodic response mechanisms has also been performed in other species. For example, a new locus *E9*, which conditions the time to flowering and maturity, has been identified in soybean (*Glycine max*) using classical genetic methods [22]. In addition to photoperiod, flowering time is also controlled by temperature and genotype-by-environment interactions. A previous study was performed to evaluate variations in flowering time and the responses of flowering time to ambient temperature and photoperiod in *V. faba*, and the results showed significant variation in responses to both ambient temperature and photoperiod [23]. Interestingly, a linear model was established to assess the relative contributions of the temperature and the photoperiod during the development of field-grown forage legumes [24]. It was found that temperature (accumulated temperature) was a very important factor limiting flowering in *V. sativa*, and the rate of progress to flowering was accelerated by warmer temperatures. Normal plant growth is always regulated by a series of plant hormones, and at least five kinds of plant endogenous hormones have been shown to influence the opening of plant flowers, including ethylene (ETH), jasmonic acids (JAs), auxin (IAA), gibberellic acids (GAs), and abscisic acid (ABA) [25,26]. Previous studies indicated that applying exogenous jasmonate and ABA delays the flowering time in *Arabidopsis* [27,28,29].

As of now, more than 300 functional genes are known to control flowering time in *A. thaliana* [30]. For example, *FLOWERING LOCUS T* (*FT*), *CONSTANS* (*CO*), *SUPPRESSOR OF OVEREXPRESSION OF CONSTANS 1* (*SOC1*), *GI*, *SHORT VEGETATIVE PHASE* (*SVP*), and *LEAFY* (*LFY*) are the key integrator genes in the flowering regulatory network [31]. As a central regulatory gene, *FT* can be influenced by different regulatory signals and the florigen protein moves from the leaves of *Arabidopsis* to the shoot apical meristem; this induces the expression of the *SOC1* gene, which influences the expression of other key genes in this flowering-regulating network, promoting flowering [32,33,34,35]. Moreover, transcription factors play a vital role in the plant flowering regulatory network. For example, the overexpression of *SlbHLH22* helps to control flowering time, accelerates fruit ripening, and produces more ethylene-producing phenotypes in tomato [36]. The MADS-box gene family is one of the most important verified flowering gene families because its members play a key role in reproductive development [37,38]. For example, the ectopic overexpression of *CsMADS02* caused earlier flowering in *Arabidopsis* [39]. In addition, some of the above flowering-related genes have high functional conservation in different species. Previous research has shown that *FT*, *SOC1*, *GI*, *SVP*, and *LFY* genes can regulate the flowering time of legumes, such as *G. max*, *Medicago truncatula* and *M. sativa* [40,41,42,43,44,45,46]. However, there are no related reports on the flowering regulatory genes in *V. sativa*.

**Figure 1:**
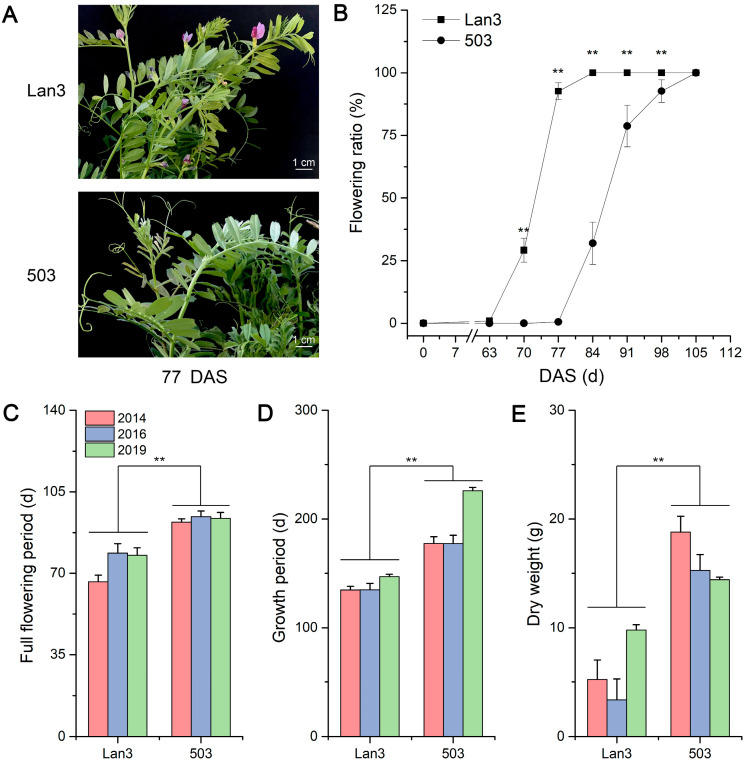
Agronomic characteristics of two *V. sativa* accessions. (**A**) The phenotypes of two *V. sativa* accessions 77 DAS. (**B**) Flowering ratio of two *V. sativa* accessions during the whole growth period. (**C**) Full flowering periods of two *V. sativa* accessions from three years of field trials. (**D**) Growth periods of two *V. sativa* accessions from three years of field trials. (**E**) Dry weights of two *V. sativa* accessions from three years of field trials. Asterisks above the bars indicate significant differences at the 0.01 level according to Duncan’s multiple range test. Scale bar = 1 cm.

RNA-Seq is an efficient and powerful tool for profiling the complete gene space of any organism, and it is used to study the flowering regulation mechanism in many plants, such as *M. sativa*, *Gossypium hirsutum*, *Paeonia suffruticosa*, and *Hemerocallis citrina* [1,30,47,48]. Metabolomics is the characterization of the metabolites that are present in an organism based on gas chromatography–mass spectrometry (GC-MS), liquid chromatography–mass spectrometry (LC-MS), and nuclear magnetic resonance platforms, and it is divided into four categories: the target analysis of metabolites, metabolic profiling, metabolomics, and metabolic fingerprint analysis [49]. To identify the flowering regulation mechanism of common vetch, we selected one early-flowering (Lan3) and one late-flowering (503) variety; collected shoot samples (shoot tip length = 10 cm) at 63, 70, and 77 days after sowing (DAS) because significant differences in flowering ratio between Lan3 and 503 appeared at these three time points (Figure 1A,B); and analyzed their transcript and metabolic dynamics. This study will promote breeding research in common vetch.

## 2. Results

### 2.1. Flowering Dynamics and the Dry Weight of V. sativa

As shown in Figure 1A,B, Lan3 began to blossom after 63 DAS, and its full-bloom stage appeared 77 DAS, but the bloom stage of 503 was two weeks after that of Lan3. We found that the full flowering period, the growth period, and the dry weight of Lan3 were significantly less than those of 503 in these three years (Figure 1C–E). Moreover, siliques of Lan3 appeared before 77 DAS (Figure S1), but new flowers have always appeared in the leaf axils at the top of the stem, because the flower of *V. sativa* is an infinite inflorescence.

### 2.2. De Novo Assembly of Transcriptome and the Functional Annotation of Unigenes

In order to further understand the molecular mechanism that is responsible for flowering in *V. sativa* at the transcriptional level, a total of 18 cDNA libraries were designed for Illumina sequencing using the shoot samples (shoot tip length = 10 cm) from Lan3 and 503 taken 63, 70, and 77 DAS (Figure S1). Ultimately, 431.42 million high-quality clean reads were obtained from the 18 libraries, and the average GC content and base of each sample were 43.66% and 7167.78 M, respectively. The Q30 ranged from 93.78% to 94.70%, with an average of 94.33%. Finally, 88,777 unigenes were assembled, with an average and N50 length of 794.56 and 1482 bp, respectively. Bowtie2 software was used to map the clean reads of each library onto the assembled unigenes. Overall, the average mapped reads and the mapped ratio were 20.93 million and 87.34% for all the libraries, respectively (Table S1).

All the unigenes were annotated into five public databases: NR, GO, KOG, COG, and KEGG. Of these 88,777 unigenes, 43,717 (49.24%), 28,734 (32.37%), 35,193 (39.64%), 16,346 (18.41%), and 19,554 (22.03%) were successfully annotated in the NR, GO, KOG, COG, and KEGG databases, respectively (Figure S2). A total of 7698 (8.67%) unigenes were annotated in all five databases, and 49,371 (55.61%) unigenes were annotated in at least one database.

### 2.3. qRT-PCR Verification

To verify the reliability and reproducibility of the RNA-Seq analysis, eight unigenes were randomly selected for qRT-PCR validation (Table S2). As a result, the expression of these selected unigenes in our transcriptome data was generally consistent with the qRT-PCR results, indicating that our RNA-Seq data were reliable (Figure S3).

### 2.4. Identification and Expression Pattern Analysis of DEGs

**Figure 2:**
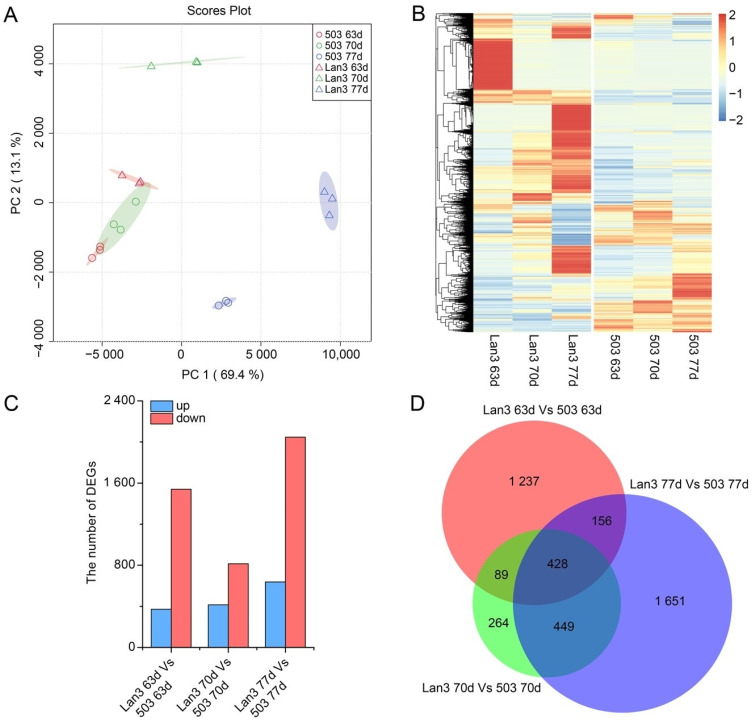
Summary of the differentially expressed genes. (**A**) PCA score plot. (**B**) Heat map of all DEGs. (**C**) A summary of the numbers of up- and down-regulated DEGs 63, 70, and 77 DAS. (**D**) The Venn diagram represents the number of overlapping DEGs between Lan3 and 503.

The FPKM values from each sample library were collected and analyzed to investigate the changes in gene expression. A PCA plot showed the high reproducibility of the biological replicates (Figure 2A). Differential expression analyses revealed that 4274 unigenes were differently expressed between Lan3 and 503 during the flowering period, and 1912, 1230, and 2684 DEGs were obtained 63, 70, and 77 DAS, respectively. These DEGs were divided into two categories according to their expression patterns, namely up-regulated and down-regulated transcripts, and the number of down-regulated DEGs was higher than that of the up-regulated DEGs at all time points (Figure 2B,C). In addition, 428 unigenes were differently expressed between Lan3 and 503 at these three time points (Figure 2D).

### 2.5. GO Functional Analysis

**Figure 3:**
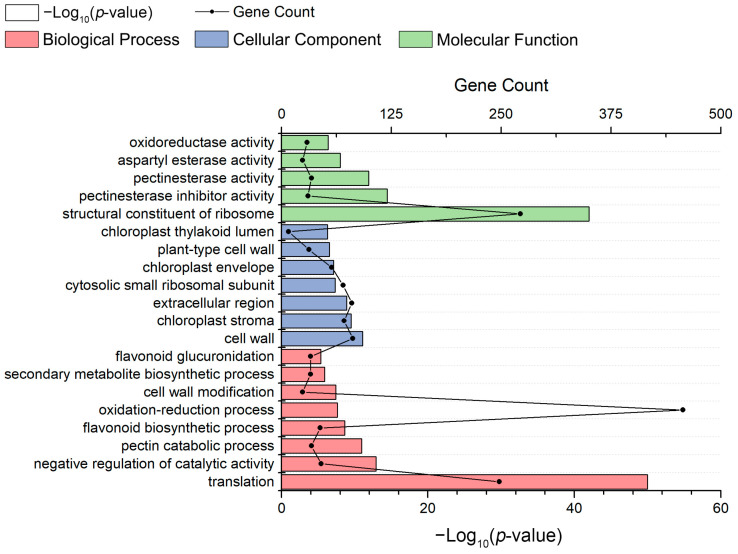
GO enrichment analysis of the top 20 most strongly represented categories. The names of the GO categories are listed along the *y*-axis. The degree of GO enrichment is represented by the −log_10_(*p*-value) (column chart) and the number of transcripts (line chart) enriched in each category.

Under the cellular component (CC) category, “cell” was the largest group (1215; 44.6%), followed by “cell part” (1206; 44.3%), “membrane” (963; 35.4%), and “organelle” (779; 28.6%). In the molecular function (MF) group, the DEGs associated with “catalytic activity” (1409; 51.7%) and “binding” (1188; 43.6%) represented the most abundant categories. Among the top three GO terms in the biological process (BP) group, “metabolic process” (1528; 56.1%), “cellular process” (1366; 50.2%), and “biological regulation” (445; 16.3%) were more enriched than other terms. Furthermore, GO enrichment (top 20) analysis was performed on the DEGs with a *p*-value cut-off of 0.05 to identify the significantly enriched GO terms among the DEGs. The results of eight BP, five MF, and seven CC categories were considered significantly enriched among the DEGs (Figure 3). Among the top 20 GO terms, “translation”, “structural constituent of ribosome”, and “cell wall” were the most significantly enriched categories in BP, MF, and CC, respectively.

### 2.6. Identification of TFs

Among the 4274 DEGs, 176 were found to belong to 23 TF families (Figure S5), and the members of the MYB family (40) were the most abundant, followed by the bHLH (28), MADS-box (19), Homeobox (15), and ZBTB (12) families. Additionally, the expression levels of 176 TFs were investigated. Accordingly, these TFs can be divided into two groups, up-regulation and down-regulation, and most of the TFs showed opposite expression patterns between Lan3 and 503 (Figure S6). These results indicate that these TFs may be involved in the flowering regulation process in *V. sativa*.

### 2.7. WGCNA of Flowering Regulation Genes

**Figure 4:**
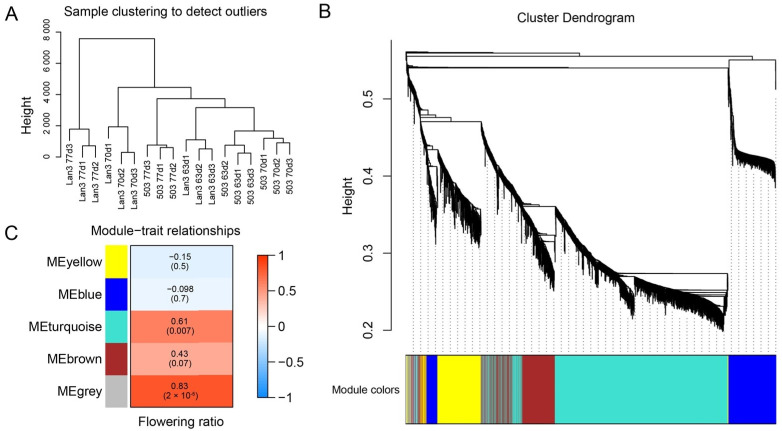
Weighted gene co-expression network analysis (WGCNA) of key genes related to flowering in *V. sativa*. (**A**) Hierarchical clustering plot of all samples. (**B**) Cluster dendrogram of all the DEGs between Lan3 and 503. Each of the genes is represented by a tree leaf, while each of the five modules is represented by a major tree branch. (**C**) Relationships between each module and the flowering ratio. The left panel shows five modules (yellow, blue, turquoise, brown, and gray); the right panel is a color scale for module trait correlations and ranges from −1 to 1.

A total of 4274 DEGs between Lan3 and 503 were obtained in the present study. A hierarchical clustering plot showed that the three biological replicates of each group were compactly gathered together and that there were no outliers (Figure 4A). To further explore the key genes regulating flowering in *V. sativa*, all the DEGs were used to construct the weighted gene co-expression network using WGCNA. Figure 4B depicts a hierarchical cluster tree with five modules. The five modules were labeled according to color: blue, brown, yellow, turquoise, and gray. Of these five modules, the turquoise module has the maximum number of genes (1624), followed by the blue (483), brown (475), yellow (408), and gray (55) modules. In addition, the correlation between the modules and the flowering ratio was analyzed, and the correlation coefficients ranged from −0.15 to 0.83. Among the five modules, the gray module had the highest correlation coefficient (0.83) and showed significant associations (*p* < 0.001) with the flowering ratio (Figure 4C). Table S3 shows the details of the members of the gray module.

### 2.8. Metabolic Characteristics of the Early and Late Flowering Genotype

**Figure 5:**
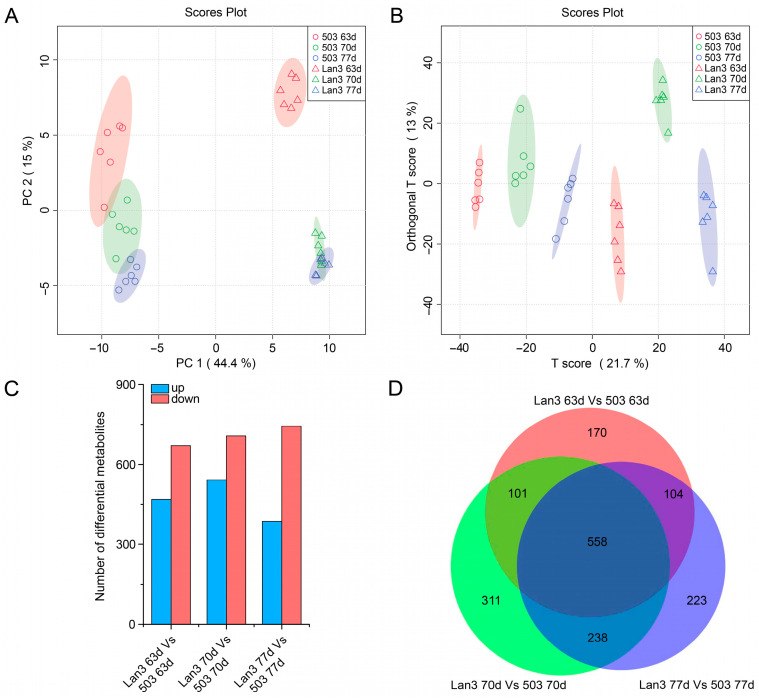
Summary of the differential metabolites between Lan3 and 503. (**A**) Principal component analysis score plot. (**B**) Orthogonal partial least squares discrimination analysis score plot. (**C**) A summary of the numbers of up- and down-regulated differential metabolites 63, 70, and 77 DAS. (**D**) The Venn diagram represents the number of overlapping differential metabolites between Lan3 and 503.

Untargeted LC-MS analysis detected 1705 differential metabolites, and the PCA (PCA1 and PCA2 represented a total of 59.4% of all data) of the genotypes and flowering periods clearly distinguished between the behavior of Lan3 and 503 (Figure 5A). Similar results were obtained by the orthogonal partial least squares discrimination analysis (OPLS-DA) (Figure 5B). Moreover, at 63, 70, and 77 DAS, 933, 1208, and 1123 differential metabolites were obtained between Lan3 and 503, respectively, and 558 differential metabolites were common to these three time points (Figure 5C,D). Among these 1705 differential metabolites, 259 metabolites were identified and enriched to 73 KEGG pathways (Tables S4 and S5).

### 2.9. Integrative Analysis of DEGs and Differential Metabolites

**Figure 6:**
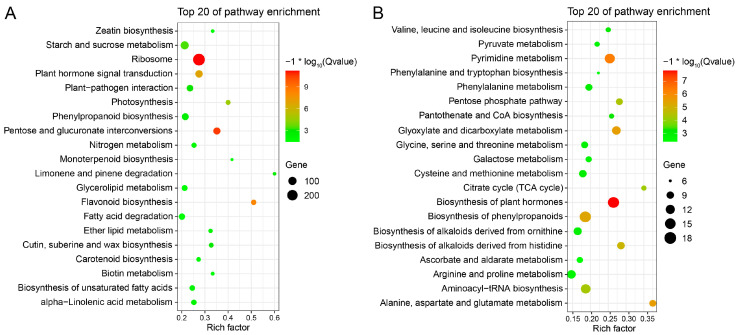
Scatterplot of enriched KEGG pathways for the DEGs (**A**) and differential metabolites (**B**) between Lan3 and 503. Only the top 20 most strongly represented pathways are displayed. The enrichment factor is the ratio of the total number of annotated genes to the DEG number in a certain pathway. The color of the dots represents the range of the −log_10_(Qvalue).

KEGG pathway enrichment analysis was performed to annotate the biological functions of the DEGs and the differential metabolites between Lan3 and 503 using the KOBAS v3.0 and MBRole 2.0 websites, respectively. Of these 4274 DEGs, 964 were annotated in the KEGG databases. From the KEGG enrichment analysis, “ribosome”, “pentose and glucuronate interconversions”, “flavonoid biosynthesis”, and “plant hormone signal transduction” were the most significantly over-represented pathways (Figure 6A). Moreover, the “biosynthesis of plant hormones”, “pyrimidine metabolism”, “glyoxylate and dicarboxylate metabolism”, and “alanine, aspartate and glutamate metabolism” pathways were the most highly represented terms in 1705 differential metabolites (Figure 6B).

**Figure 7:**
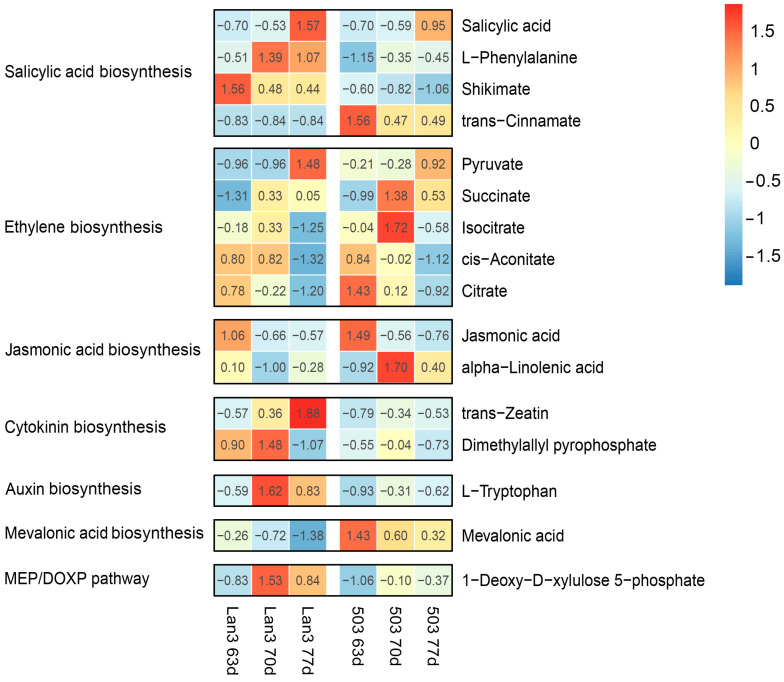
The contents of 16 differential metabolites related to the biosynthesis of plant hormones in Lan3 and 503. The colors indicate the abundance of metabolites calculated as log_2_(Value).

**Figure 8:**
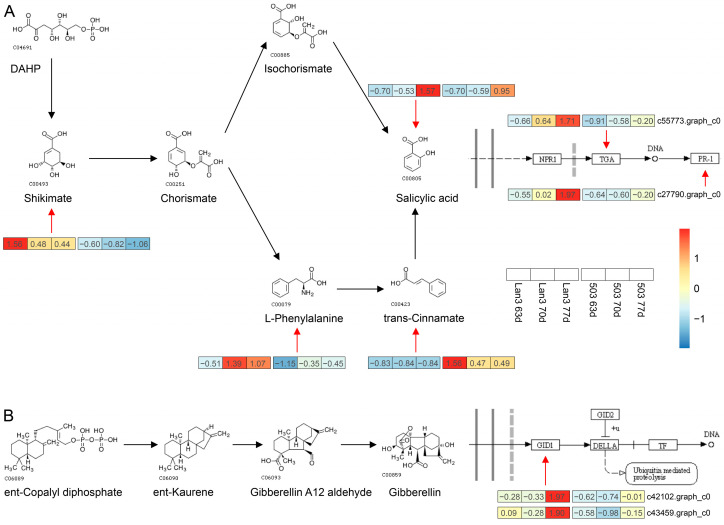
Biosynthesis and signal transduction of salicylic acid (**A**) and gibberellin (**B**) in *V. sativa*. Heatmap of transcript levels for genes and contents of the metabolites involved in the biosynthesis and signal transduction of salicylic acid and gibberellin.

A total of 16 differential metabolites and 65 DEGs were obtained in the “biosynthesis of plant hormones” pathway and “plant hormone signal transduction” pathway, respectively (Figure 7 and Table S6). Among the 16 differential metabolites, four metabolites were found to belong to the “salicylic acid biosynthesis” pathway; five metabolites were found to belong to the “ethylene biosynthesis” pathway; and the other metabolites were found to belong to the “jasmonic acid biosynthesis” (two), “cytokinin biosynthesis” (two), “auxin biosynthesis” (one), “mevalonic acid biosynthesis” (one), and the “MEP/DOXP pathway” (one). According to the “biosynthesis of plant hormones” (map01070) and the “plant hormone signal transduction” (map04075) pathways in the KEGG database [50], we drew the biosynthesis and signal transduction pathways of salicylic acid and gibberellin in *V. sativa* using the differential metabolites and DEGs data obtained in the present study. As shown in Figure 8A, the contents of four metabolites (shikimate, L-phenylalanine, trans-cinnamate, and salicylic acid) related to “salicylic acid biosynthesis” showed significant differences between Lan3 and 503. Moreover, the Pearson correlation coefficients between 16 differential metabolites and 65 DEGs were calculated, and a significant correlation was found between the four metabolites related to “salicylic acid biosynthesis” and most of the DEGs (Figure S7). Additionally, the expression levels of two DEGs (c55773.graph_c0 and c27790.graph_c0) related to “salicylic acid signal transduction” in Lan3 were higher than those in 503 (Figure 8A), and two DEGs (c42102.graph_c0 and c43459.graph_c0) related to “gibberellin signal transduction” showed similar trends (Figure 8B).

### 2.10. Variation of Salicylic Acid Content in Various V. sativa Accessions

**Figure 9:**
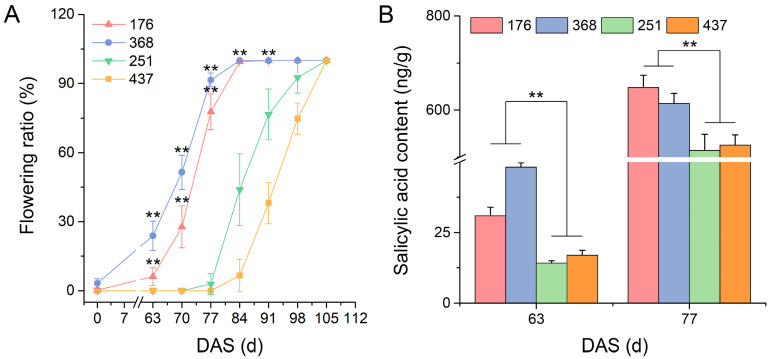
The flowering ratio (**A**) and salicylic acid content (**B**) in various *V. sativa* accessions. The asterisks above the bars for early-flowering accessions (176 and 368) indicate significant differences at the 0.01 level compared to late-flowering accessions (251 and 437) according to Duncan’s multiple range test.

As shown in Figure 9A, the *V. sativa* accessions 176 and 368 began to blossom 63 DAS, and their full-bloom stage appeared 84 DAS, while 251 and 437 began to blossom 77–84 DAS. In conclusion, the bloom stages of 251 and 437 were two weeks later than those of 176 and 368. In order to further verify the variation in the salicylic acid content in early- and late-flowering *V. sativa* accessions (Figure S8), the shoot samples (shoot tip length = 10 cm) of 176, 368, 251, and 437 were collected 63 and 77 DAS, and the salicylic acid contents of these samples were determined. The salicylic acid content of each *V. sativa* accession significantly increased during the flowering process (Figure 9B). Significantly, the salicylic acid content in the early-flowering accessions were higher than those in late-flowering accessions at these two time points, which is consistent with the metabolome sequencing results of Lan3 and 503.

### 2.11. Flowering-Related Genes in V. sativa

**Figure 10:**
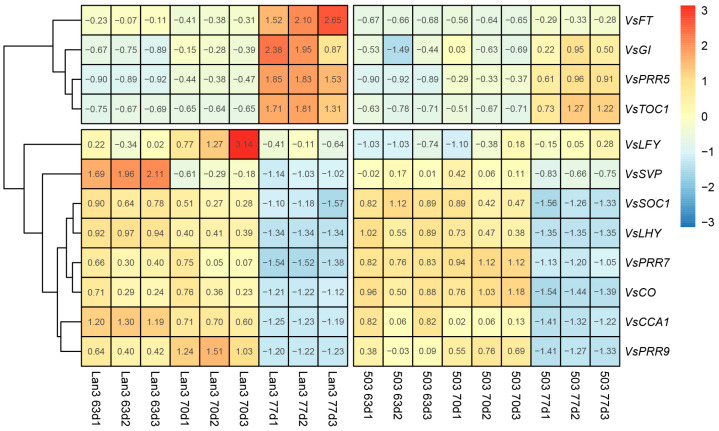
The expression patterns of 12 reported flowering-related genes in Lan3 and 503. The colors indicate the transcript levels calculated as log_2_(FPKM).

In the present study, we investigated the expression patterns of 12 reported flowering-related genes in Lan3 and 503 (Figure 10). As a result, these flowering-related genes can be divided into two groups, including eight early expressed genes (*VsLFY*, *VsSVP*, *VsSOC1*, *VsLHY*, *VsPRR7*, *VsCO*, *VsCCA1*, and *VsPRR9*) and four late expressed genes (*VsFT*, *VsGI*, *VsPRR5*, and *VsTOC1*). Moreover, the expression levels of six genes (*VsFT*, *VsGI*, *VsPRR5*, *VsTOC1*, *VsLFY*, and *VsSVP*) in Lan3 were significantly higher than those in 503, indicating that these six flowering-related genes may be involved in the flowering regulation process in *V. sativa*.

## 3. Discussion

### 3.1. Flowering Time Affects the Yield of V. sativa

Different from agricultural production, the above-ground part of the plant is the harvest target of forage production, so increasing biomass yield is the principal goal of most breeding programs in forages. Floral transition is a major developmental switch in the life of flowering plants and dictates whether photosynthetic products should be invested in vegetative growth or reproductive development [51]. Therefore, the delayed flowering time is one of the principal approaches for achieving increased biomass yield. Switchgrass is a perennial C_4_ grass that is undergoing development as a dedicated biomass feedstock for conversion to bioenergy, and its biomass yield is strongly influenced by delayed flowering, averaging +0.03 to 0.13 Mg ha^−1^ for each day’s delay in the anthesis date [52]. A previous study reported that the flowering time shows a significant positive association with days to pod initiation, days to maturity, plant height, and the biomass of chickpea [53], and similar results appear in the present study. Three years of field trials have shown that the growth period and the dry weight of early-flowering accession (Lan3) are significantly less than those of late-flowering accession (503) (Figure 1), indicating that the flowering time can affect the yield of *V. sativa*.

As of now, many functional genes are known to control the flowering time in *A. thaliana*. For example, *PRR* genes encode the clock-associated transcriptional repressors that act redundantly. A study demonstrated that the constitutive expression of engineered *PRR5* (*PRR5-VP*) can increase biomass and abiotic stress tolerance, and concomitant analyses of the relative growth rate, flowering time, and photosynthetic activity suggested that the increased biomass of *PRR5-VP* plants is mostly due to late flowering [54]. As a high-quality legume forage, *V. sativa* is cultivated worldwide, but there are no related reports on the flowering regulatory genes in *V. sativa*. In the present study, we found that the expression level of *VsPRR5* increased significantly at 77 DAS and was significantly higher in Lan3 than it was in 503 (Figure 10), indicating that *VsPRR5* may regulate the flowering time in *V. sativa*.

### 3.2. TFs Involved in the Flowering Process in V. sativa

An increasing amount of evidence has indicated that many TF families, such as MYB, bHLH, Dof, and MADS-box, are involved in the plant flowering process [36,37,38,39]. In the present study, a total of 23 TF families were obtained from the 4274 DEGs between the Lan3 and 503 accessions (Figure S5), and the MYB, bHLH, and MADS-box families have the most members. The role of MYB proteins in plant development is essential; they function in diverse biological processes, including in stress and defense responses and in seed and floral development [55]. Under long-day conditions, the overexpression of the wheat MYB-related transcription factor *TaMYB72* in rice shortened the flowering time by approximately 12 days [56]. The chrysanthemum gene *CmMYB2*, an R2R3 MYB transcription factor, was confirmed to interact with CmBBX24, a zinc-finger transcription factor that is known to regulate flowering through its influence on gibberellin synthesis; plants that have been engineered to overexpress *CmMYB2* flowered earlier than wild-type plants, while those in which *CmMYB2* was suppressed flowered later [57]. Moreover, bHLH and MADS-box gene family members play a key role in plant reproductive development. For example, a previous study showed the early flowering phenotype in the *Arabidopsis cib2* mutant and the complementation of the early flowering phenotype by *35S::AcCIB2*-GFP [58]. The ectopic overexpression of one *Phyllostachys edulis* MADS-box gene, *PeMADS5*, triggered earlier flowering in *Arabidopsis* and the development of an aberrant flower phenotype [59]. We found that the expression levels of most MYB, bHLH, and MADS-box TFs are significantly different between Lan3 and 503 accessions and that most of the members of these three gene families have high transcriptional levels in early-flowering accession (Figure S9). These results indicate that MYB, bHLH, and MADS-box TFs may regulate flowering in *V. sativa*. It is important to note that the early flowering plant (Lan3) had siliques by the last sampling date (Figure S1), which may lead to the presence of some genes related to pod development in the DEGs. Therefore, some TFs obtained in the present study may also regulate the pod development of *V. sativa*.

### 3.3. Role of Plant Hormones in the Flowering Process in V. sativa

Flowering is regulated by a complex network of genes that integrate multiple environmental cues and endogenous signals so that flowering occurs at the right time; hormone regulation, signaling, and homeostasis are important in this process [27]. The KEGG enrichment analyses of the DEGs and differential metabolites showed that plant hormone biosynthesis and signal transduction are significantly enriched pathways in the flowering process in *V. sativa* (Figure 6A,B), and the results were similar to those for *Jasminum sambac* and *Solanum lycopersicum* [60,61]. Combined with the KEGG enrichment analysis results of the DEGs and the differential metabolites between the Lan3 and 503 accessions, we can speculate that the synthesis and the signal transduction of plant hormones play an important role in the flowering process in *V. sativa*. Additionally, shoot samples (shoot tip length = 10 cm) of Lan3 and 503 were collected 63, 70, and 77 DAS for the integrative analyses of the transcriptomes and metabolomes in the present study, but siliques of Lan3 have inevitably appeared before 77 DAS due to the flower of *V. sativa* is an infinite inflorescence, which may lead to the presence of some genes or metabolites related to pod development in the plant hormone biosynthesis and signal transduction pathways. GAs regulate seed development, stem elongation, leaf expansion, and floral transition, and they play an important role in one of the major flowering pathways. In the current model of GA signaling, GA binds to a soluble GID1 receptor, which in turn binds to the DELLA repressor protein. Three putative *GID1* genes (*PhGID1A*, *PhGID1B*, and *PhGID1C*) encoding GA receptors were isolated from petunia, and the virus-induced gene silencing (VIGS) of these genes results in stunted growth, dark-green leaves, and late flowering [62]. In our study, the expression levels of two *V. sativa GID1* genes (c42102.graph_c0 and c43459.graph_c0) in the early-flowering accession (Lan3) were higher than those in the later flowering accession (503) (Figure 8B), which suggests that these *GID1* genes are involved in the flowering process in *V. sativa*.

Several previous studies have reported that SA can efficiently induce flowering in plants, such as *Lemna gibba*, *Sinningia speciose*, and *Gazania rigens* [63,64,65]. Four metabolites related to salicylic acid biosynthesis were obtained in the present study, and the contents of three metabolites (shikimate, L-phenylalanine, and SA) were higher in Lan3 than they were in 503 (Figure 8A). Significantly, similar results also appeared in the two other early-flowering (176 and 368) and late-flowering (251 and 437) accessions (Figure 9B). The genes *TGA* and *PR-1*, which regulate the development of flower organs and flowering, were involved in the SA signal transduction pathway. For example, the *Arabidopsis tga7* mutant displayed a delayed-flowering phenotype under both long-day and short-day conditions, and plants lacking *TGA9* and *TGA10* have defects in male gametogenesis [66,67]. All the *PR-1* genes in plants appear to be inducible by SA, and many *PR-1* genes are constitutively expressed in the roots or floral tissues, which is indicative of a role in plant development [68]. In this study, the *TGA* and *PR-1* genes in *V. sativa* showed a higher transcription level in the early-flowering accession (Figure 8A). These results suggest that the salicylic acid pathway may be involved in flowering regulation in *V. sativa*.

## 4. Materials and Methods

### 4.1. Plant Materials and Sample Collection

Six accessions of *V. sativa* with different flowering characteristics were used in the present study, including three early-flowering accessions (Lan3, 176, and 368) and three late-flowering accessions (503, 251, and 437) (Figures S1 and S8). Lan3 was cultivated by our laboratory, and 503 (PI 284459), 176 (PI 179111), 368 (PI 383798), 251 (PI 251200), and 437 (PI 420420) were selected from 222 *V. sativa* accessions provided by the National Plant Germplasm System of the United States after three years of field experiments. These accessions were grown in the Yuzhong Experimental Station of Lanzhou University (35°57′ N; 104°09′ E; 1720 m above sea level), Lanzhou, China. The mean annual precipitation and mean annual temperature in this area are 350 mm and 6.7 °C, respectively, and the day lengths range from 9.7 to 14.5 h (Figure S10).

The flowering ratios of these six accessions were monitored after sowing in 2019, and the full flowering period, the growth period, and the dry weight of Lan3 and 503 were measured for three years (2014, 2016, and 2019). After sowing, the number of open flowers per plant is recorded weekly, and the flowering ratio refers to the ratio of the number of flowers that have opened to the number of all flowers on a single plant. The full flowering period refers to the number of days from sowing to when the flowering ratio reaches 80%; the growth period refers to the number of days from emergence to the withering and yellowing of the above-ground parts of a single plant; the dry weight refers to the weight of the above-ground parts (stubble height = 5 cm) of a single plant that are in the mature stage after drying. Three biological replicates were performed for the above indicators. All the data were subjected to one-way analysis of variance (ANOVA) for a completely randomized design using SPSS 19.0 (IBM, Armonk, New York, NY, USA). Differences among means were evaluated by Duncan’s multiple range test at *p* < 0.05. Additionally, three and six individual plants from Lan3 and 503 were selected in 2019 for transcriptome (each sample containing three biological replicates) and metabolome (each sample containing six biological replicates) sequencing, respectively. According to the flowering ratio, the shoot samples (shoot tip length = 10 cm) from Lan3 and 503, including two fully expanded compound leaves and three incompletely expanded compound leaves, were collected 63, 70, and 77 DAS (Figure S1). These samples were immediately frozen in liquid nitrogen and stored at −80 °C.

### 4.2. RNA Extraction and Library Construction for Transcriptome Analysis

The total RNA of each sample was isolated using the Trizol method (Sangon Biotech, Shanghai, China) according to the manufacturer’s instructions. The RNA concentration was determined using a NanoDrop ND8000 spectrophotometer (Thermo Scientific, Waltham, MA, USA), and the RNA integrity was detected using an RNA Nano 6000 Assay Kit with an Agilent Bioanalyzer 2100 system (Agilent Technologies, Santa Clara, CA, USA). In the present study, a total of 18 cDNA libraries were constructed using an mRNA-Seq Sample Preparation Kit according to the manufacturer’s instructions (NEBNext^®^Ultra™ RNA Library Prep Kit for Illumina^®^, NEB, Ipswich, MA, USA). Poly(A)-containing mRNA was enriched via oligo magnetic adsorption, and the enriched mRNA was fragmented. Then, double-strand cDNA (dscDNA) was obtained by reverse transcription with an N6 random primer. Furthermore, purification, adapter ligation, and PCR amplification were performed according to RNA-Seq protocols. These libraries were sequenced on the Illumina Hiseq 2000 platform created by the Beijing Biomarker Institution (Beijing, China).

### 4.3. Sequence Filtering, De Novo Assembly, and Functional Annotation

Sequencing reads were filtered to obtain high-quality clean reads because raw reads additionally contain low-quality, adaptor-polluted reads, and a high content of unknown base (N) reads. Afterward, transcriptome assembly was accomplished using Trinity with the min_kmer_cov set to 2 by default and all other parameters set to default [69], and the unigene function was annotated based on the following databases: NCBI non-redundant protein sequences (NR), Eukaryotic Orthologous Group (KOG), Clusters of Orthologous Groups of proteins (COG), Kyoto Encyclopedia of Genes and Genomes (KEGG), and Gene Ontology (GO).

### 4.4. Quantitative Real-Time PCR Analysis

To verify the RNA-Seq results, eight unigenes were selected for a quantitative real-time PCR (qRT-PCR) test. qRT-PCR was conducted using 2 × SG Fast qPCR Master Mix (Sangon Biotech, Shanghai, China) on the CFX96 Touch™ Real-Time PCR Detection System (Bio-Rad, Hercules, CA, USA), and the results were analyzed with CFX Manager software (Bio-Rad, Hercules, CA, USA). The qRT-PCR was programmed as follows: 95 °C for 3 min and 39 cycles of 95 °C for 10 s and 55 °C for 30 s. The specific primers were designed using Primer6 software (Premier Biosoft International, Palo Alto, CA, USA), as shown in Table S2. Three technical replicates were performed for each sample, and the relative expression levels were normalized for *Vsactin* gene expression and calculated using the 2^−ΔΔCt^ method [70].

### 4.5. Differential Expression of Unigenes

The transcript expression levels were identified by the RSEM software package and calculated by the fragments per kilobase of transcript per million mapped transcript (FPKM) method for each sample. Based on the average FPKM values in each sample, the differential expression between the different samples from Lan3 and 503 was assessed using the DESeq R package. In order to identify significant differentially expressed genes (DEGs), absolute fold change values of ≥2 and adjusted *p*-values of ≤0.05 were set as the threshold.

### 4.6. DEG Analysis

In the present study, the cluster analysis and the expression pattern assessment of the DEGs were performed using the Pheatmap R package. To annotate the biological functions of the DEGs, GO enrichment analysis and KEGG pathway enrichment analysis were conducted using agriGO 2.0 and KOBAS 3.0 [71,72], respectively. To predict the transcription factors (TFs) in the DEGs, the open reading frame (ORF) of each unigene was examined using Getorf software [73], and then aligned to the TF domains using Hmmsearch [74]. The TFs were then identified according to the regulations described in the PlntfDB database [75]. In addition, weighted gene co-expression network analysis (WGCNA) was performed using R package WGCNA V4.0.3 to further explore the flowering-related genes in *V. sativa* [76]. An applicable soft-thresholding power based on the scale-free topology criterion was employed to transform the correlation matrix to a signed weighted adjacency matrix by calculating the Pearson correlation between all the genes. The resulting adjacency matrix was used to calculate the topological overlap matrix (TOM). Subsequently, all the genes were hierarchically clustered based on similarity using a dynamic tree cut to form a module with a minimum module size threshold of 20 genes [77].

### 4.7. Untargeted LC-MS

Widely targeted metabolome analysis was performed by the Beijing Biomarker Institution (Beijing, China) according to their standard procedures. Briefly, six replicate samples of the shoot tissues (shoot tip length = 10 cm) from Lan3 and 503 were collected 63, 70, and 77 DAS. Frozen 50 mg samples were weighed, and 1000 μL of precooled extraction buffer (400 μL methanol, 400 μL acetonitrile, and 200 μL H_2_O) was added. Then, these samples were treated with a 45 Hz grinding instrument for four mins and were ultrasonicated for five mins (in an ice water bath). The supernatant was collected in tubes after centrifugation (12,000 rpm, 4 °C, 15 min), and the extract was dried in a vacuum concentrator. To the dried metabolite, 150 μL of extraction solution (75 μL acetonitrile and 75 μL H_2_O) was added, and the resulting solution was vortexed for 30 s and placed in an ice bath ultrasound for 10 min. Then, 120 μL of the supernatant was carefully removed in an injection bottle after centrifugation (12,000 rpm, 4 °C, 15 min). The LC-MS system for metabolomics analysis consisted of an Acquity I-Class PLUS ultra-high-performance liquid chromatography in tandem with a Xevo G2-XS QTof high-resolution mass spectrometer. The raw data were collected using MassLynx V4.2 software (Waters Corporation, Milford, MA, USA), and peak extraction and peak alignment were performed using Progenesis QI software (Progenesis, La Jolla, CA, USA). Afterward, online databases such as METLIN and the self-built database of the Beijing Biomarker Institution were used to identify the metabolites.

Principal component analysis (PCA) and orthogonal partial least squares discrimination analysis (OPLS-DA) were carried out using the online software MetaboAnalyst 5.0 [78]. Differential metabolites were set to have a fold change >1, a *p*-value <0.05, and a VIP >1. In order to annotate the biological functions of the differential metabolites, KEGG pathway enrichment analysis was conducted using MBRole 2.0 [79]. Moreover, the Pearson correlation coefficient between the DEGs and differential metabolites was calculated, and the correlation heat map was drawn in the program R.

### 4.8. Determination of the Salicylic Acid Content

Shoot samples (shoot tip length = 10 cm) were collected from four other accessions (176, 368, 251, and 437) 63 and 77 DAS in 2019, and these samples were immediately frozen in liquid nitrogen and stored at −80 °C. The salicylic acid content of these samples was determined according to the manufacturer’s instructions (SaA-4-Q, Komin, Suzhou, China). The data are presented as the means ± the standard deviation of at least three independent experiments, and statistical analyses were conducted using the one-way analysis of variance test with the spss19.0 program.

## 5. Conclusions

Flowering is a central event in the plant life cycle and an important determinant of harvesting time. Three years of field trials showed that the growth period and the dry weight of early-flowering accession (Lan3) are significantly less than those of late-flowering accession (503). To reveal the molecular regulatory mechanism of the flowering process in *V. sativa*, the shoot samples (shoot tip length = 10 cm) of these two accessions were collected 63, 70, and 77 DAS for integrative analyses of the transcriptomes and metabolomes and 4274 DEGs and 259 differential metabolites between Lan3 and 503 were obtained. Among all the DEGs, 40 MYB, 28 bHLH, and 19 MADS-box TFs were identified, and most of these TFs demonstrated high transcription levels in the early-flowering accession. Moreover, 55 key regulatory genes for the flowering in *V. sativa* were identified by WGCNA. KEGG enrichment showed that the biosynthesis and signal transduction of plant hormones may play an important role in the flowering process in *V. sativa*, and the contents of three metabolites related to salicylic acid biosynthesis and the transcription levels of two DEGs related to salicylic acid signal transduction in Lan3 were higher than they were in 503. Further verification in various accessions indicated that salicylic acid biosynthesis and signal transduction may be involved in the flowering regulation process in *V. sativa*. This in-depth elucidation of the flowering process lays the foundation for the breeding research of *V. sativa*.

## Data Availability

All sequencing reads are available in NCBI SRA: SRR17967534-SRR17967539.

## Supplementary Materials

The following supporting information can be downloaded at: https://www.mdpi.com/article/10.3390/ijms23126818/s1.
