# Supplementary material for: Integrative Analyses of Transcriptomes and Metabolomes Reveal Associated Genes and Metabolites with Flowering Regulation in Common Vetch (Vicia sativa L.)
Source: Int J Mol Sci. 2022 Jun 19;23(12):6818. doi: 10.3390/ijms23126818 (PMC9224626; doi:10.3390/ijms23126818)
Supplement: Supplementary file 1 [file ijms-23-06818-s001.zip › Supplementary Figures.pdf]

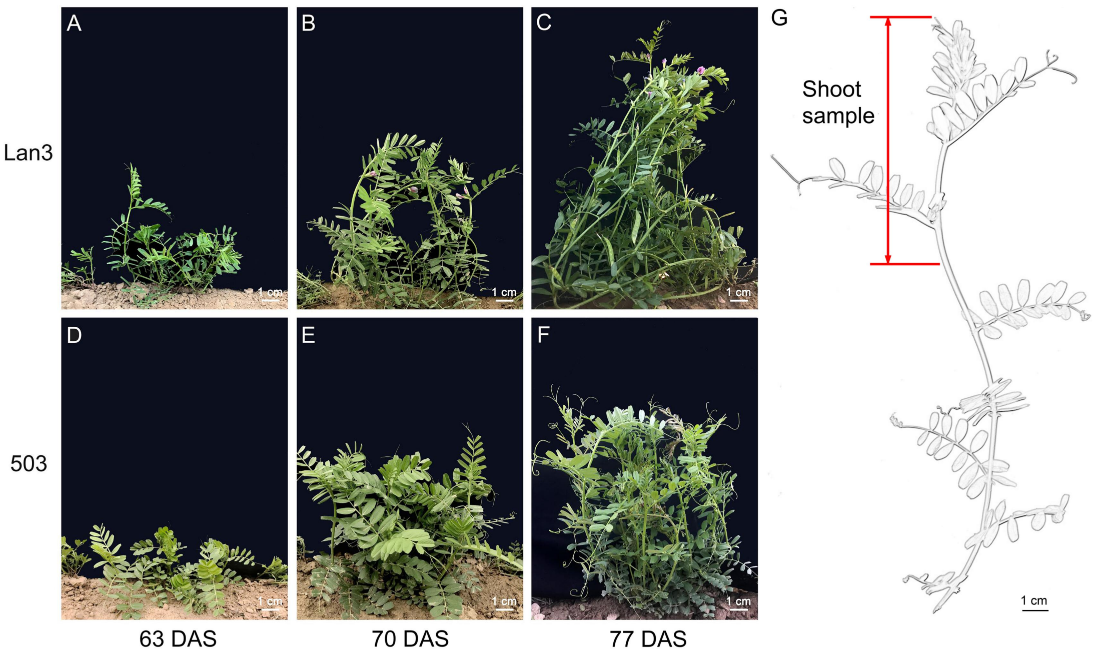

Figure S1. Two *V. sativa* accessions were used for the integrative analyses of the transcriptomes and metabolomes. Shoot samples (shoot tip length = 10 cm) of two *V. sativa* accessions were collected 63 (A and D), 70 (B and E), and 77 (C and F) DAS in 2019. The red marked area in the schematic diagram (G) is the sampling position of the shoot sample. Scale bar = 1 cm.

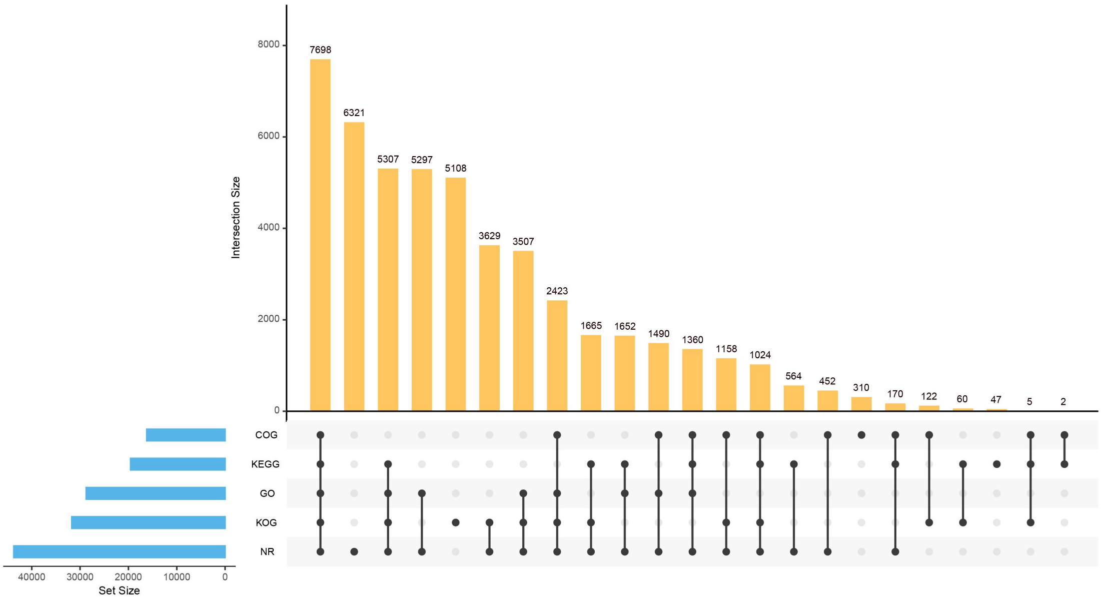

Figure S2. The Venn diagram represents the number of overlapping unigenes annotated in five public databases.

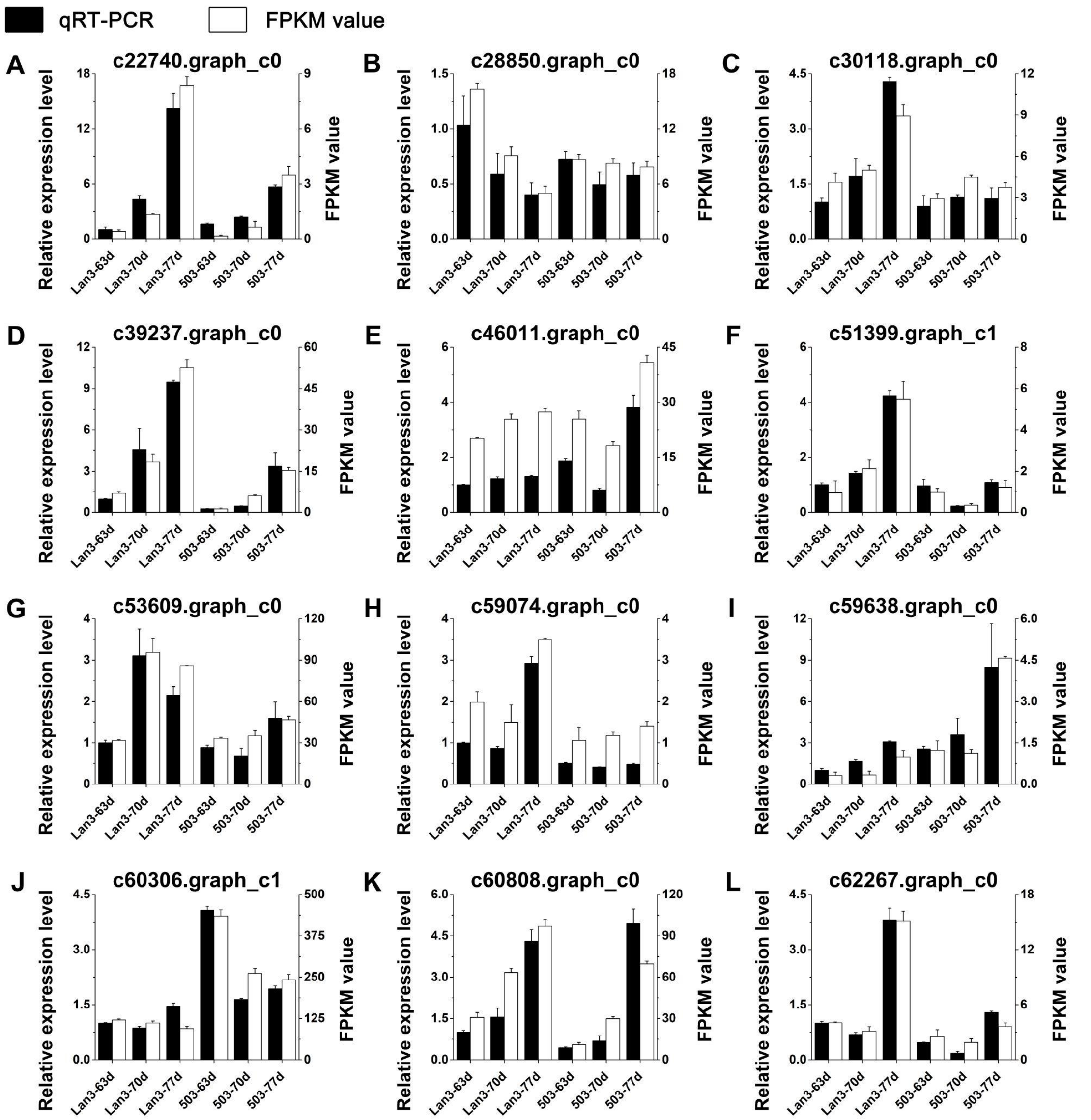

Figure S3. The expression patterns of 12 randomly selected genes, which were identified by RNA-Seq, were validated by qRT-PCR. The black bars represent the relative expression levels determined by qRT-PCR (left y-axis). The white bars indicate the transcript abundance changes based on the fragments per kilobase per million fragments mapped (FPKM) values of the RNA-Seq analysis (right y-axis). The error bars indicate the standard errors of the means (n = 3).

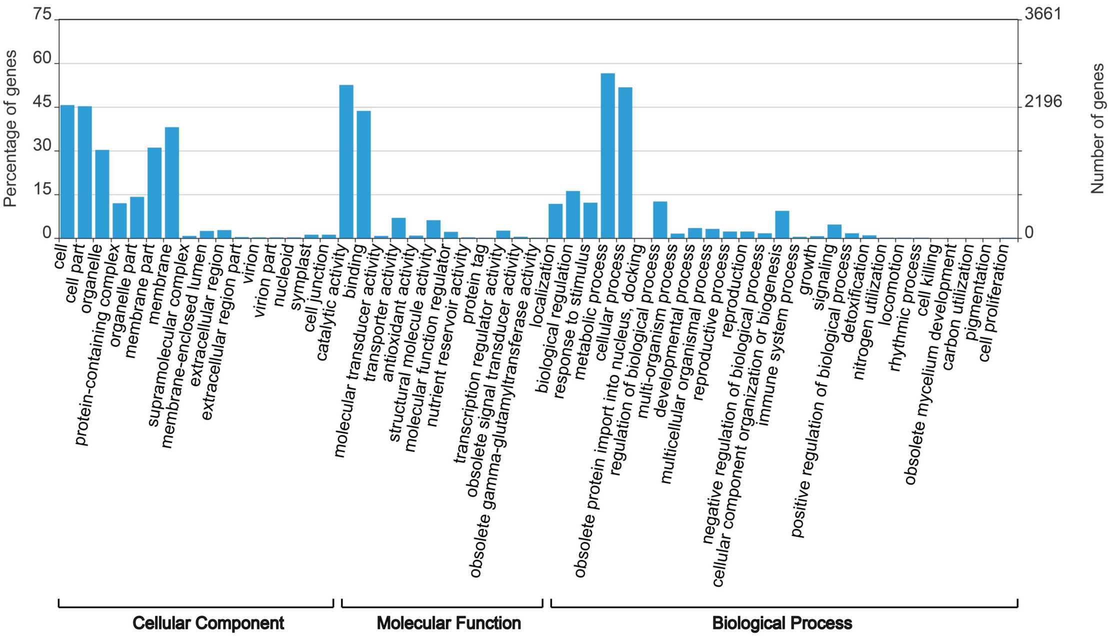

Figure S4. Histogram of GO terms assigned to DEGs between Lan3 and 503. The DEGs are categorized into three main groups: cellular components (CCs), molecular functions (MFs), and biological processes (BPs).

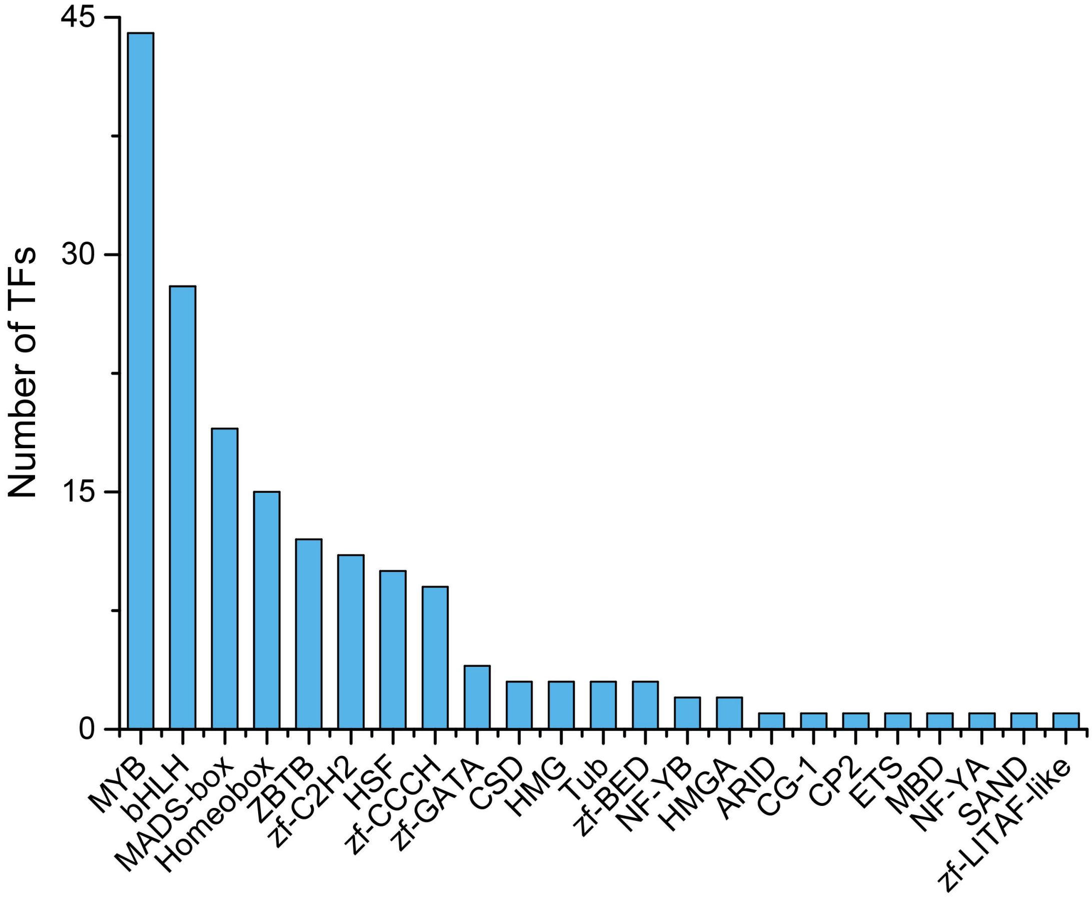

Figure S5. Distribution of transcription factors in DEGs between Lan3 and 503.

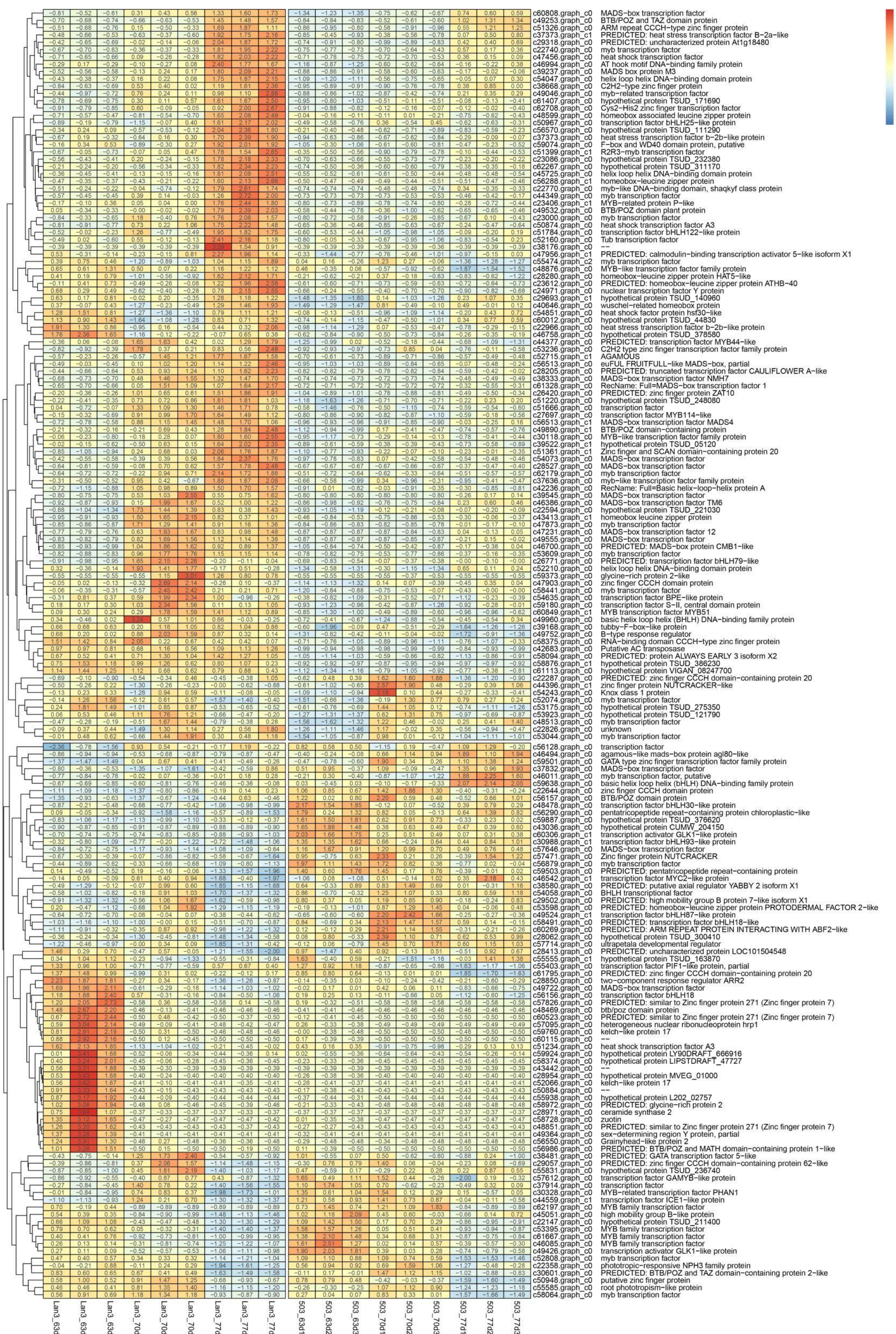

Figure S6. Heat map of the 176 transcription factors identified in the DEGs. The colors indicate the abundance of transcripts calculated as log_2(FPKM). Further information about each gene is provided on the right.

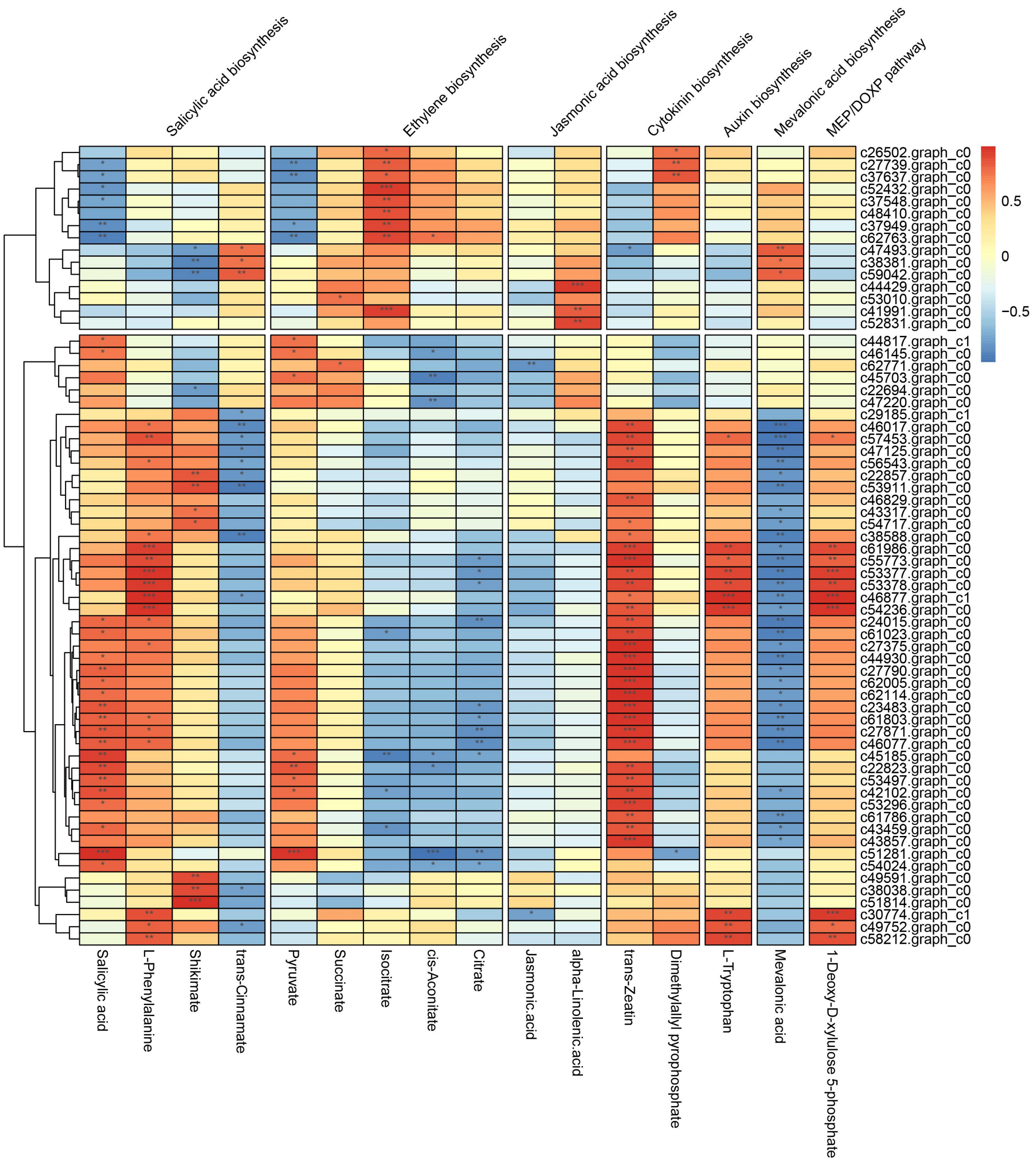

Figure S7. Relationships between differential metabolites related to the biosynthesis of plant hormones and DEGs related to plant hormone signal transduction. “***”, “**”, and “*” indicate significant differences at the 0.001, 0.01, and 0.05 levels, respectively.

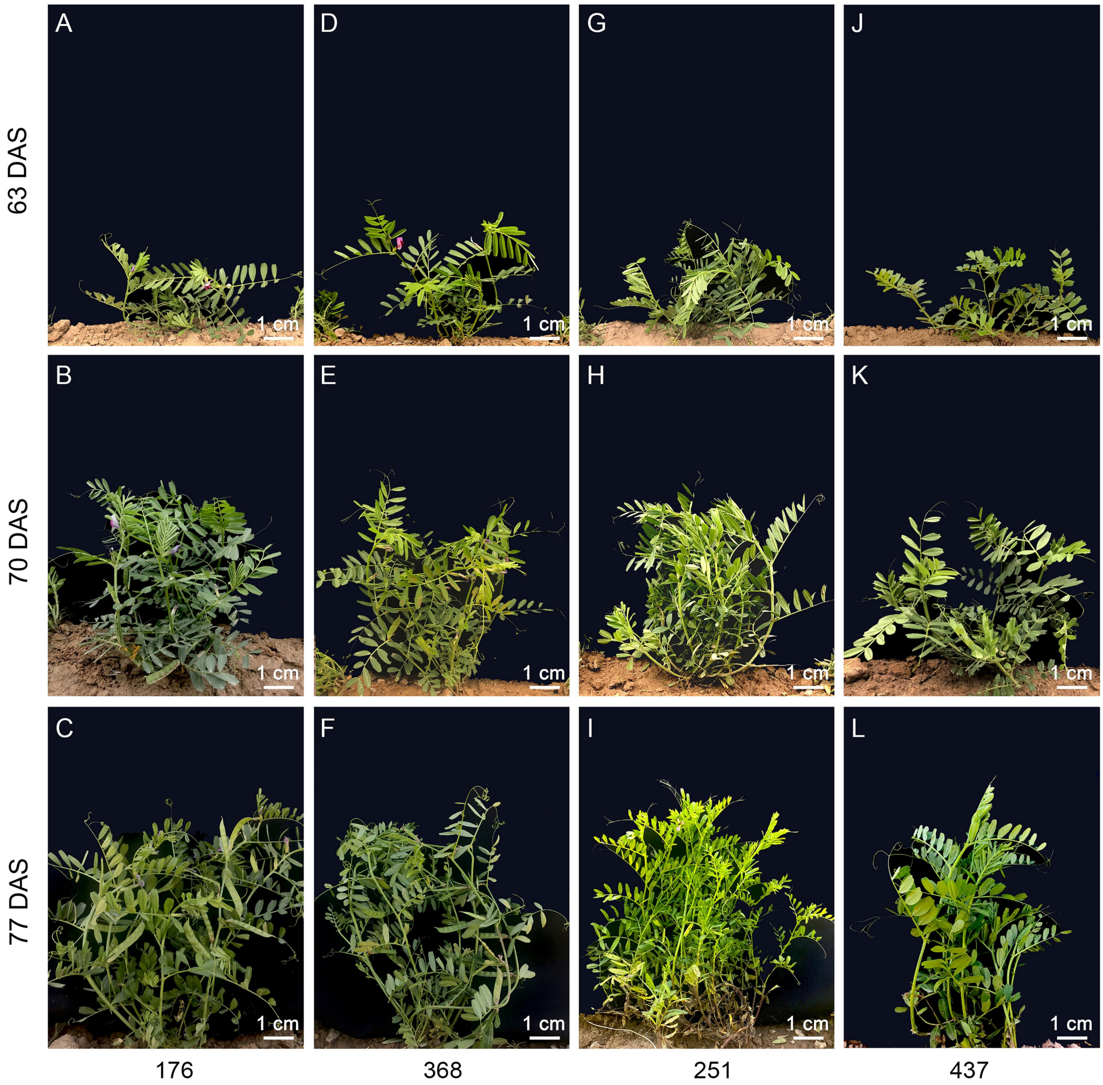

Figure S8. Four *V. sativa* accessions for the determination of the salicylic acid content. 176 and 368 are early-flowering accessions; 251 and 437 are late-flowering accessions. We observed the phenotypes of four *V. sativa* accessions 63 (A, D, G, and J), 70 (B, E, H, and K), and 77 (C, F, I, and L) DAS in 2019. Scale bar = 1 cm.

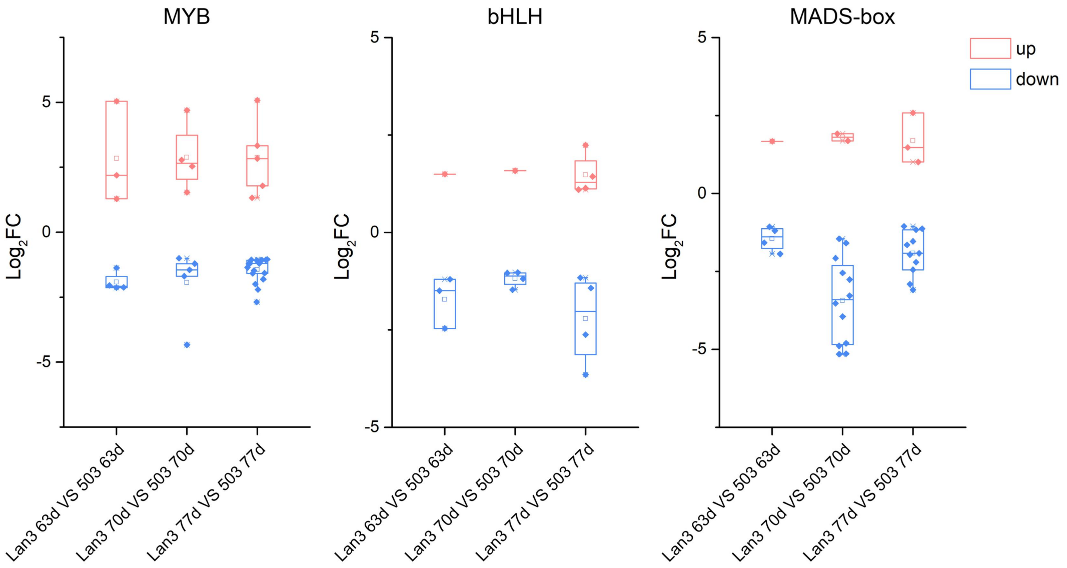

Figure S9. The expression level distribution of the MYB, bHLH, and MADS-box transcription factors in Lan3 and 503.

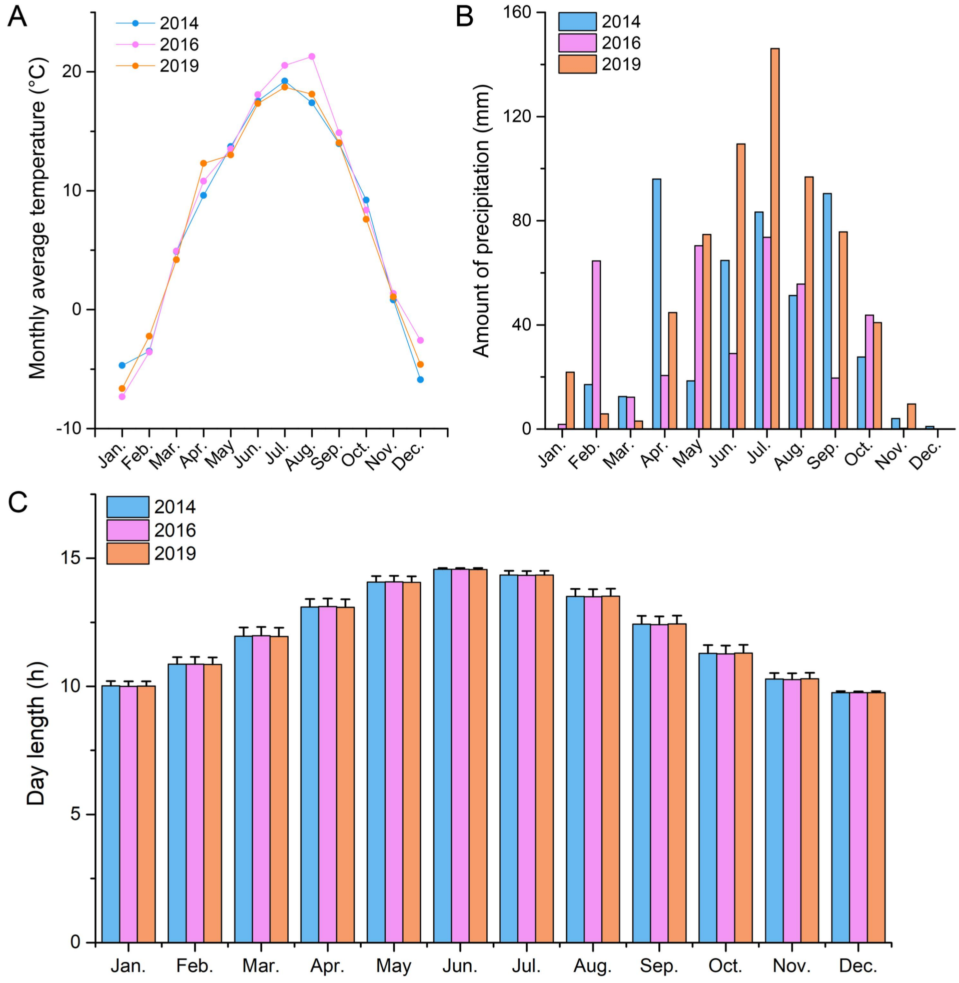

Figure S10. Monthly average temperatures (A), amounts of precipitation (B), and day lengths (C) in 2014, 2016, and 2019 in Yuzhong, Gansu, China. The above data are from two meteorological websites (http://www.wheata.cn/; https://sunsetsunrisetime.com/).
